# COVID-19: The question of genetic diversity and therapeutic intervention approaches

**DOI:** 10.1590/1678-4685-GMB-2020-0452

**Published:** 2022-04-08

**Authors:** David Livingstone Alves Figueiredo, João Paulo Bianchi Ximenez, Fábio Rodrigues Ferreira Seiva, Carolina Panis, Rafael dos Santos Bezerra, Adriano Ferrasa, Alessandra Lourenço Cecchini, Alexandra Ivo de Medeiros, Ana Marisa Fusco Almeida, Anelisa Ramão, Angelica Beate Winter Boldt, Carla Fredrichsen Moya, Chung Man Chin, Daniel de Paula, Daniel Rech, Daniela Fiori Gradia, Danielle Malheiros, Danielle Venturini, Eliandro Reis Tavares, Emerson Carraro, Enilze Maria de Souza Fonseca Ribeiro, Evani Marques Pereira, Felipe Francisco Tuon, Franciele Aní Caovilla Follador, Glaura Scantamburlo Alves Fernandes, Hélito Volpato, Ilce Mara de Syllos Cólus, Jaqueline Carvalho de Oliveira, Jean Henrique da Silva Rodrigues, Jean Leandro dos Santos, Jeane Eliete Laguila Visentainer, Juliana Cristina Brandi, Juliana Mara Serpeloni, Juliana Sartori Bonini, Karen Brajão de Oliveira, Karine Fiorentin, Léia Carolina Lucio, Ligia Carla Faccin-Galhardi, Lirane Elize Defante Ferreto, Lucy Megumi Yamauchi Lioni, Marcia Edilaine Lopes Consolaro, Marcelo Ricardo Vicari, Marcos Abdo Arbex, Marcos Pileggi, Maria Angelica Ehara Watanabe, Maria Antônia Ramos Costa, Maria José S. Mendes Giannini, Marla Karine Amarante, Najeh Maissar Khalil, Quirino Alves de Lima, Roberto H. Herai, Roberta Losi Guembarovski, Rogério N. Shinsato, Rubiana Mara Mainardes, Silvana Giuliatti, Sueli Fumie Yamada-Ogatta, Viviane Knuppel de Quadros Gerber, Wander Rogério Pavanelli, Weber Claudio da Silva, Maria Luiza Petzl-Erler, Valeria Valente, Christiane Pienna Soares, Luciane Regina Cavalli, Wilson Araujo Silva

**Affiliations:** 1 Universidade Estadual do Centro-Oeste do Paraná (UNICENTRO), Departamento de Medicina, Guarapuava, PR, Brazil; 2 Instituto para Pesquisa do Câncer (IPEC), Guarapuava, PR, Brazil.; 3 Universidade de São Paulo, Faculdade de Ciências Farmacêuticas de Ribeirão Preto, Departamento de Análises Clínicas, Toxicologia e Ciência de Alimentos, Ribeirão Preto, SP, Brazil.; 4 Universidade Estadual do Norte do Paraná (UENP), Centro de Ciências Biológicas, Bandeirantes, PR, Brazil.; 5 Universidade Estadual do Oeste do Paraná, Francisco Beltrão, PR, Brazil.; 6 Universidade de São Paulo, Faculdade de Medicina de Ribeirão Preto, Hemocentro Regional de Ribeirão Preto, Ribeirão Preto, SP, Brazil.; 7 Universidade Estadual de Ponta Grossa, Ponta Grossa, Programa de Pós Graduação em Computação Aplicada, Ponta Grossa, PR, Brazil.; 8 Universidade Estadual de Londrina, Departamento de Patologia Geral, Londrina, PR, Brazil.; 9 Universidade Federal do Paraná, Programa de Pós-Graduação em Genética, Departamento de Genética, Curitiba, PR, Brazil.; 10 Universidade Estadual do Centro-Oeste do Paraná (UNICENTRO), Departamento de Medicina Veterinária, Guarapuava, PR, Brazil.; 11 Universidade Estadual Paulista (UNESP), Faculdade de Ciências Farmacêuticas, Departamento de Fármacos e Medicamentos, Araraquara, SP, Brazil.; 12 União das Faculdades dos Grandes Lagos (UNILAGO), Centro de Pesquisa Avançada em Medicina, São José do Rio Preto, SP, Brazil.; 13 Universidade Estadual do Centro-Oeste do Paraná (UNICENTRO), Departamento de Farmácia, Guarapuava, PR, Brazil.; 14 Universidade Estadual do Oeste do Paraná (UNIOESTE), Hospital do Câncer Francisco Beltrão, Laboratório de Biologia de Tumores, Francisco Beltrão, PR, Brazil.; 15 Universidade Estadual de Londrina, Centro de Ciências da Saúde, Departamento de patologia, clínica e toxicologia, Laboratório de bioquímica clínica, Londrina, PR, Brazil.; 16 Universidade Estadual de Londrina, Departamento de Microbiologia, Centro de Ciências Biológicas, Londrina, PR, Brazil.; 17 Universidade Estadual do Centro-Oeste do Paraná (UNICENTRO), Laboratório de Virologia Clínica, Guarapuava, PR, Brazil.; 18 Universidade Estadual do Centro-Oeste do Paraná (UNICENTRO), Departamento de Enfermagem, Guarapuava, PR, Brazil.; 19 Universidade Católica do Paraná, Laboratório de Doenças Infecciosas Emergentes, Pontifícia Curitiba, PR, Brazil.; 20 Universidade Estadual do Oeste do Paraná, Departamento de Ciências da Vida, Programa de Pós-Graduação em Ciências Aplicadas à Saúde, Francisco Beltrão, PR, Brazil.; 21 Universidade Estadual de Londrina, Departamento de Biologia Geral, Londrina, PR, Brazil.; 22 Universidade Estadual do Paraná (UNESPAR), Faculdade de Ciências Biológicas, Centro de Ciências Humanas e Educação, Paranavaí, PR, Brazil.; 23 Universidade do Estado de São Paulo (UNESP), Faculdade de Ciências Farmacêuticas, Departamento de Fármacos e Medicamentos, Araraquara, SP, Brazil.; 24 Universidade Estadual de Maringá, Laboratório de Imunogenética, Maringá, PR, Brazil.; 25 Universidade Estadual Paulista (UNESP), Faculdade de Ciências Farmacêuticas, Departamento de Análises Clínicas, Araraquara, SP, Brazil.; 26 Universidade Estadual do Centro-Oeste do Paraná (UNICENTRO), Laboratório de Neuropsicofarmacologia, Guarapuava, PR, Brazil.; 27 Universidade Estadual de Londrina, Departamento de Ciências Patológicas, Centro de Ciências Biológicas, Laboratório de Genética Molecular e Imunologia, Londrina, PR, Brazil.; 28 Faculdades Pequeno Príncipe, Instituto de Pesquisa Pelé Pequeno Príncipe, Curitiba, PR, Brazil.; 29 Universidade Estadual do Oeste do Paraná, Programa de Pós-Graduação em Ciências Aplicadas à Saúde, Centro de Ciências da Saúde, Francisco Beltrão, PR, Brazil.; 30 Universidade Estadual de Maringá, Departamento de Análises Clínicas e Biomedicina, Maringá, PR, Brazil.; 31 Universidade Estadual de Ponta Grossa, Departamento de Biologia e Genética Estrutural e Molecular, Ponta Grossa, PR, Brazil.; 32 Universidade de Araraquara, Faculdade de Medicina, Área temática de Pneumologia, Araraquara, SP, Brazil.; 33 Universidade Estadual de Londrina, Departamento de Ciências Patológicas, Centro de Ciências Biológicas, Laboratório de Imunologia, Londrina, PR, Brazil.; 34 Universidade do Estado do Paraná, Colegiada de Enfermagem, Curitiba, PR, Brazil.; 35 Universidade Católica do Paraná (PUCPR), Faculdade de Medicina, Programa de Pós-Graduação em Ciências da Saúde, Laboratório Experimental Multiusuário, Curitiba, PR, Brazil.; 36 Universitário Católico Salesiano Auxilium (UNISALESIANO), Faculdade de Medicina, Centro Araçatuba, SP, Brazil.; 37 Universidade Estadual de Londrina, Laboratório de Imunoparasitologia de Doenças Negligenciadas e Câncer, Londrina, PR, Brazil.; 38 Faculdade de Medicina de Ribeirão Preto, Centro de Terapia Celular (CEPID/FAPESP), Ribeirão Preto, SP, Brazil.; 39 Instituto Nacional de Ciência e Tecnologia em Células-Tronco e Terapia Celular (INCT/CNPq), Ribeirão Preto, SP, Brazil.; 40 Universidade de São Paulo, Faculdade de Medicina de Ribeirão Preto, Departamento de Genética, Ribeirão Preto, SP, Brazil.; 41 Novos Arranjos de Pesquisa e Inovação - Genômica (NAPI-Genômica), Fundação Araucária, PR, Brazil.; 42 Universidade Estadual do Centro-Oeste do Paraná (UNICENTRO), Departamento de Ciências Biológicas, Guarapuava, PR, Brazil.

**Keywords:** COVID-19, therapeutic interventions, global health treat, virus diversity

## Abstract

Coronavirus disease 2019 (COVID-19), caused by the Severe Acute Respiratory Syndrome Coronavirus type 2 (SARS-CoV-2), is the largest pandemic in modern history with very high infection rates and considerable mortality. The disease, which emerged in China’s Wuhan province, had its first reported case on December 29, 2019, and spread rapidly worldwide. On March 11, 2020, the World Health Organization (WHO) declared the COVID-19 outbreak a pandemic and global health emergency. Since the outbreak, efforts to develop COVID-19 vaccines, engineer new drugs, and evaluate existing ones for drug repurposing have been intensively undertaken to find ways to control this pandemic. COVID-19 therapeutic strategies aim to impair molecular pathways involved in the virus entrance and replication or interfere in the patients’ overreaction and immunopathology. Moreover, nanotechnology could be an approach to boost the activity of new drugs. Several COVID-19 vaccine candidates have received emergency-use or full authorization in one or more countries, and others are being developed and tested. This review assesses the different strategies currently proposed to control COVID-19 and the issues or limitations imposed on some approaches by the human and viral genetic variability.

## Introduction

The scientific community considers the COVID-19 caused by the new coronavirus SARS-CoV-2 as the deadliest pandemic in recent human history. SARS-CoV-2 is a virus of the family *Coronaviridae* of the genus *Betacoronavirus*, with the subgenus *Sarbecovirus*. Many coronaviruses have been identified in several animal species, of which six infect human hosts, including the severe acute respiratory syndrome-related coronavirus (SARS-CoV-1) and the Middle East respiratory syndrome coronavirus (MERS-CoV) (Dietz *et al*., 2020; Guo *et al*., 2020). The genome of the new coronavirus was fully sequenced (NCBI Reference Sequence: NC_045512.2) (Wang *et al*., 2020). Its sequence presents about 82% identity to the bat SARS-like coronavirus WIV1 (bat SL-CoV-WIV1, GenBank: KF367457.1), and more than 85% identity with the bat SARS-like coronavirus ZC45 (bat SL-CoV-ZC45, GenBank: MG772933.1) (Li X *et al*., 2020; Yu *et al*., 2020).

SARS-Cov-2 is an enveloped, non-segmented positive-sense RNA virus with prominent stick-shaped protruding particles in their outer membrane (Peng *et al*., 2020; Yin *et al*., 2020). Similar to SARS-CoV-1 and MERS-CoV, the SARS-CoV-2 genome encodes nonstructural proteins (NSPs, such as 3-chymotrypsin-like protease, papain-like protease, helicase, and RNA-dependent RNA polymerase), structural and accessory proteins (Li Q *et al*., 2020). Among NSPs, NSP1 is the first protein of the polyprotein of SARS CoV-2 and a leader protein, which acts as a potent inhibitor of gene expression of the virus carrier (Huang *et al*., 2011). Nonstructural protein 2 (NSP2) binds two other host proteins, prohibitin 1 and prohibitin 2 (PHB1 and PHB2), disrupting the host cell environment (Cornillez-Ty *et al*., 2009). NSP3, the papain-like proteinase protein, has multiple functions and is considered the most important protease of the virus (Báez-Santos *et al*., 2015). 

This new coronavirus has four major structural proteins: the spike (S), small envelope (E), and membrane (M) glycoproteins, and nucleocapsid (N) protein, besides several accessory proteins. The trimeric S protein is indispensable for virus-cell receptor interactions during viral entry (Lu *et al*., 2020; Walls *et al*., 2020). SARS-CoV-2 targets cells through the S protein, which binds to the human angiotensin-converting enzyme 2 (ACE2) receptor and employs the cellular serine protease TMPRSS2 for S protein priming (Datta *et al*., 2020; Hoffmann *et al*., 2020; Letko *et al*., 2020; Tai *et al*., 2020). Notably, the ACE2 receptor is expressed in various tissues and organ systems throughout the body, including the central nervous system, gastrointestinal system, heart, lung, testes, and kidney (Baig *et al*., 2020; Zhang *et al*., 2021). In fact, in addition to oropharyngeal swabs, the viral RNA has also been detected in blood, urine, facial/anal swabs, semen, and vaginal secretion, suggesting other potential means of transmission (Peng *et al*., 2020). Ultimately, the S protein binding to the ACE2 receptor triggers a cascade of events leading to the fusion and releasing of the viral RNA genome into the host cell. The nonstructural proteins are subsequently synthesized to encode the viral replicase-transcriptase complex. The viral RNA is then synthesized by RNA-dependent RNA polymerase (Chen Y *et al*., 2020; Letko *et al*., 2020). Further, when the virus is in the cytosol, the non-structural viral proteins (nsp) 1-16 are produced and catalyze replication of the viral RNA genome, and inhibition of the host’s innate immune response (Thiel *et al*., 2003; Gildenhuys, 2020). The Mpro or NSP5 protease mediates the cleavage of the viral replicative proteins, RNA-dependent RNA polymerase (RpRp) and helicase (HEL) (Ziebuhr *et al*., 2000).

SARS-CoV-2 has one of the hardest outer protective shells among all coronaviruses. This feature is believed to result in more stable viral particles, resulting in greater resilience in body fluids (Goh *et al*., 2020). Another relevant and recurrent challenge imposed by this pandemic, is the emergence of distinct new-high transmissible variants around the globe; so far, five variants of concern (VOC) have already been identified, B.1.1.7, detected first in the UK, B.1.351, initially detected in South Africa, B.1.1.28.1 (also known as P.1), first detected in Brazilian travellers in Japan, and more recently, B.1.427 and B.1.429, identified in USA (Centers for Disease Control and Prevention, CDC, 2021). These new variants prevent the body’s immune response by selecting and excluding pieces of the virus’s genetic sequence. In this sense, there was a need for further studies on the pathogenicity and replication of SARS-CoV-2. 

Regarding the diagnostic tools, the highly specific reverse-transcriptase polymerase-chain reaction (RT-PCR) technology is the gold standard test for COVID-19 and data from epidemiological evidence and clinical manifestations combined with radiological images, such as computer tomography (CT), also have critical diagnostic value for COVID-19 (Li X *et al*., 2020). Clinically, COVID-19 presents a myriad of possible symptoms and outcomes, from asymptomatic carriage, flu-like symptoms including cough, fever, general weakness, myalgia, pneumonia-like characteristics, and respiratory failure requiring mechanical ventilation (Itelman *et al*., 2020). Although there are studies that point out that COVID-19 manifests itself as a respiratory tract infection, rising data have been demonstrating that COVID-19 should be treated as a systemic disease, involving the most diverse systems of the human body, such as gastrointestinal, cardiovascular, respiratory, renal, neurological, immunological and hematopoietic (Driggin *et al*., 2020; Mehta *et al*., 2020). 

The transmission patterns of SARS-CoV-2 and its pathogenicity motivates the scientific community to work against the clock to improve the diagnostic, preventive and therapeutic management of the disease, and to identify the genetic risk factors. There is no current evidence to recommend any specific anti-SARS-CoV-2 treatment for patients with suspected or confirmed COVID-19. Diverse therapeutic interventions are being evaluated in clinical trials, and new approaches are being proposed regarding pharmacological therapy for COVID-19 (Saber-Ayad *et al*., 2020). 

**Figure 1 -:**
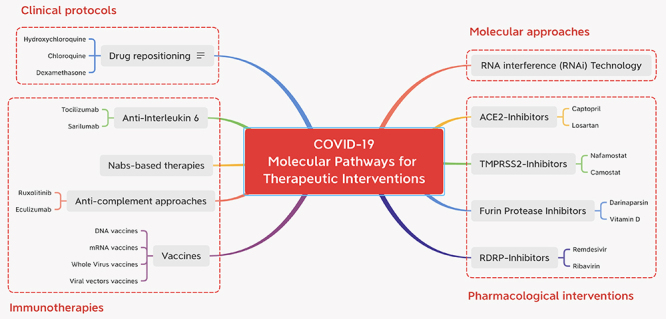
Main routes for therapeutic intervention of the COVID-19. The article discusses four approaches that are being used in an attempt to treat patients with severe clinical evolution.

In the light of the actual scenario, the repurposing of drugs, the development of novel effective immunotherapies, and safe and effective long-lasting vaccines against the SARS-CoV-2 are essential strategies for coping with this pandemic. In this review, we aim to discuss the current status of therapeutic interventions against COVID-19 (Figure 1), highlighting them from a mechanistic point of view considering the role of microRNAs, viral characteristics, and host genetic determinants, as well as the feasibility of the available drugs. A review of the current research on these topics may help guide strategies to address the current COVID-19 pandemic and prepare us for future challenges.

## Genetic basis of COVID-19 clinical phenotypes

The clinical heterogeneity observed in COVID-19 most likely results from the interaction of the immune responses and comorbidities presented by patients. The genetic background of patients certainly plays an essential role in this regard. Genetic variants of the cellular components that allow the interaction of the viral particle with the host cell and its entry are the most obvious candidates for investigation. Moreover, many of the components of the human innate and adaptive immune responses present genetic variants that may have functional impact. The genetic variability of the SARS-CoV-2 may provide additional factors modulating the disease manifestations (Hofmann *et al*., 2004; Li W *et al*., 2005, 2007; Cao Y *et al*., 2020; Pinto *et al*., 2020). Besides, hormonal factors inherent to sex can influence the risk of mortality in cases positive for SARS-CoV-2. Karlberg *et al*. (2004) studied the mortality rate from the Hong Kong SARS epidemic and observed a significant difference between men (21.9%) and women (13.2%). Coincidence or not, the *ACE2* gene is located on the X chromosome (Xp22) (Li *et al*., 2003). Oophorectomy or treatment of mice with an estrogen receptor inhibitor resulted in increased mortality in females infected with SARS-CoV-1 (Channappanavar *et al*., 2017). The other research front has concentrated efforts on the characterization of the different strains of SARS-CoV-2 to establish the viral subtypes and analyze the genetic variants associated with the different clinical phenotypes of COVID-19. In this case, the genomic regions whose products are responsible for the entry of SARS-CoV-2 in the host cells have been considered the principal candidates (Channappanavar *et al*., 2017; Benvenuto *et al*., 2020; Bezerra *et al*., 2020; Coutard *et al*., 2020; Licastro *et al*., 2020; Lu *et al*., 2020; Rehman *et al*., 2020; Sah *et al*., 2020; Shereen *et al*., 2020; Zhao *et al*., 2021). The analysis of a specific genomic signature of the SARS-CoV-2 strains can help in understanding the viral evolution since the first case reported in China (Fan *et al*., 2020). A computational tool was applied to identify and track numerous strains of SARS-CoV-2 circulating on different continents, especially those isolated from hospitalized patients, whether or not they needed intensive care and pulmonary ventilation (Zhao *et al*., 2021). The authors used a public database containing 4087 SARS-CoV-2 sequences and were able to define at least ten strains that infected patients in the United States, realizing that some of them are the same found in Asia and Europe. Such reports can help projects aiming to correlate SARS-CoV-2 strains with the clinical evolution of hospitalized patients. 

### Genetic diversity of the SARS-CoV-2

RNA viruses present higher mutation rates than DNA viruses, especially the single-stranded RNA (ssRNA) viruses, such as the SARS-CoV-2 (Peck and Lauring, 2018), although the SARS-CoV-2 and other related viruses perform proofreading during RNA replication, differently from most other RNA viruses (Romano *et al*., 2020). Data from the Global Initiative on Sharing All Influenza Data (GISAID) (Elbe and Buckland-Merrett, 2017) have indicated that the SARS-CoV-2 mutational rate (Shen Z *et al*., 2020) was similar to other coronaviruses (Eckerle *et al*., 2010; Son *et al*., 2020). The single nucleotide polymorphisms (SNPs) are the most frequent variants in the genome of the SARS-CoV-2 and are considered the leading cause of the genetic diversity and evolution of the virus, besides its virulence and transmissibility (Yin, 2020). The SNPs can be found in both coding and non-coding regions of the viral genome. SNPs located in coding regions have a high potential to contribute to the classification of new strains of SARS-CoV-2, calculate the rate of infection, and design vaccines and define effective doses for different population groups (Saha *et al*., 2020). One study carried out with virus isolates from Europe has shown that SNPs are more frequent in proteins related to viral replication (RNA polymerase) and ACE2 binding regions of the S protein. These genetic variants have been previously associated with the effectiveness of vaccines (Yin, 2020). Studies in other populations have described SNPs in the genes encoding NSP-2, and also RdRp and the S protein (Tabibzadeh *et al*., 2020).

The SARS-CoV-2 evolves *in vivo* after infection, which may affect its virulence, infectivity, and transmissibility (Shen Z *et al*., 2020). Indeed, several studies have analyzed the mutational profile of interhost and intrahost single nucleotide variants (iSNV). The analysis of large datasets has shown that SARS-CoV-2 presented a more significant proportion of G>T changes in both iSNVs and iSNPs compared to SARS-CoV-1 and MERS. Interestingly, the mutational profile of the iSNVs was more similar among SARS-CoV-2 and MERS-CoV than SARS-CoV-1 (Sapoval *et al*., 2021). Altogether, the data presented above indicated that genetic variations in the SARS-CoV-2 genome sequence could be critical to assist in the definition of the virus transmission pattern and to control the infection outbreak, as well as for epidemiological monitoring and tracking of the virus. 

The ∆382 corresponds to the deletion of the nucleotide in the position 382 which truncates the ORF7b and removes the ORF8 transcription-regulatory sequence. This variant is associated with milder illness compared to the wild-type virus, probably due to reduced cytokine release during the acute phase of the disease. The mechanism of this attenuated variant suggests that ORF8 can be a target for therapeutic intervention (Young *et al*., 2020). Conversely, SARS-CoV-2 that bears the D614G mutation in the S protein is associated with a higher case fatality rate (Becerra-Flores and Cardozo, 2020), a fact that should be considered for design of therapeutic antibodies and prognosis. 

### Genetic diversity of the human host

Viral targets in the host cells, such as the ACE2 and TMPRSS2, have been considered molecular markers to determine the genetic susceptibility or resistance to COVID-19 (Mohammadpour *et al*., 2021). Several studies have shown that the presence of polymorphisms in the *ACE2* gene can affect: (i) the modulation of intermolecular interactions with the SARS-CoV-2 S protein (Benetti *et al*., 2020; Gibson *et al*., 2020; Hussain *et al*., 2020; Lippi *et al*., 2020); (ii) the binding to the viral S protein (Li, Q *et al*., 2020; Stawiski *et al*., 2020); (iii) the structure and stabilization (Benetti *et al*., 2020), and the expression of the ACE2 receptors (Badawi, 2020; Cao Y *et al*., 2020; Delanghe *et al*., 2020). ACE2 variants usually alter the interaction between host cells and SARS-CoV-2 by showing lower affinity to the virus proteins that bind host cells’ surface, thus conferring decreased susceptibility to COVID-19 (Stawiski *et al*., 2020).

ACE2 expression differs on the basis of the biological age and sex of each individual (Goren *et al*., 2020; Ovsyannikova *et al*., 2020), and also according to the different geographic and ethnic distribution of the COVID-19 patients (McCoy *et al*.,2020; Sun *et al*., 2020). A large number of studies have described SNPs in patients of distinct countries affecting the molecular mechanisms cited above (Badawi, 2020; Benetti *et al*., 2020; Cao Y *et al*., 2020; Delanghe *et al*., 2020; Gibson *et al*., 2020; Hatami *et al*., 2020; Hussain *et al*., 2020; Lippi *et al*., 2020; Stawiski *et al*., 2020; Yamamoto N *et al*., 2020). According to Alifano *et al*. (2020), these polymorphisms could explain in part the differences currently observed in COVID-19 incidence between countries around the world, despite the globalization of exchanges and travels. A gene homologous to *ACE2*, the human *ACE1* gene that is mapped on chromosome 17, presents a polymorphic insertion (I) or deletion (D) of a 287-base pair (bp) Alu repeat sequence in intron 16 (Rieder *et al*.,1999), that has been shown to impact susceptibility to the disease as well as the frequency of recoveries and deaths (Delanghe *et al*., 2020; Hatami *et al*., 2020; Yamamoto N *et al*., 2020; Calabrese *et al*., 2021). 

Other studies have reported the association of polymorphisms in other protein cell receptors, such as the TMPRSS2 receptor (Asselta *et al*., 2020; Hou *et al*., 2020; Russo *et al*., 2020; Senapati *et al*., 2020; Torre-Fuentes *et al*., 2021), as well as in the *HLA* genes (Nguyen *et al*., 2020; Lorente *et al*., 2021; Amoroso *et al*., 2021; Warren and Birol, 2021) and *ABO* blood group locus (Ellinghaus *et al*., 2020; Amoroso *et al*., 2021; Zhao *et al*., 2021), with the risk of acquiring COVID-19. These results suggest that HLA antigens may influence SARS-CoV-2 infection and clinical evolution of COVID-19, and confirm that blood group A individuals are at greater risk of infection. In most of these studies, the variants observed were associated with the susceptibility to SARS-CoV-2 infection, as well as with the severity of the disease, such as the development of cardiovascular and respiratory complications (Ellinghaus *et al*., 2020; Hou *et al*., 2020; Amoroso *et al*., 2021; Lorente *et al*., 2021;). A review of the possible impact of genetic factors involved in the immune responses on COVID-19 can be found in Anastassopoulou *et al*. (2020). 

### Variability in the human and viral miRNA network and the control of host response to SARS-CoV-2

MicroRNAs (miRNAs), a class of non-coding small RNA molecules, are important post-transcriptional regulators that have been associated with the development of several pathologies, including the ones caused by viral infections (Maltby *et al*., 2016; Trobaugh and Klimstra, 2017; Girardi *et al*., 2018; Stolzenburg and Harris, 2018; Dutta *et al*., 2019; Tribolet *et al*., 2020). Human (host) and viral miRNAs interact with each other and although these interactions are not yet completely elucidated, it is very likely to involve the regulation of cellular processes that affect virus pathogenicity and cellular response (Totura and Baric, 2012; Bruscella *et al*., 2017). The gene network associated with host responses can result from miRNA transcriptional regulation of a subset of mRNA targets that are critical components of signaling pathways, including the WNT, INF, PIK3/AKT, MAPK, and NOTCH pathways (Barbu *et al*., 2020; Khan M *et al*., 2020). On the other hand, miRNAs from the virus can deregulate host miRNAs and facilitate the viral replication, induce the latency, prevent apoptosis, and/or cause immune evasion (Salmena *et al*., 2011; Scheel *et al*., 2016; Trobaugh and Klimstra*,* 2017; Damas *et al*., 2019; Mishra *et al*., 2020). SARS-CoV-2 genome mutations have also been reported to disrupt the binding sites of miRNAs and negatively impact the modulation of anti-virus host defenses (Rad and McLellan, 2020), as well as viral miRNA sponges that can deplete specific host miRNAs (Bartoszewski *et al*., 2020; Srivastava *et al*., 2020). 

In the infection by SARS-CoV-2, the identification of the potential virus-human miRNA-based interactions have been mostly conducted on computational miRNA prediction analysis (Arisan *et al*., 2020; Khan M *et al*., 2020; Nersisyan *et al*., 2020; Saçar and Adan, 2020; Sarma *et al*., 2020; Marchi *et al*., 2021). Based on the seed region specificity, Arisan *et al.* (2020) have compared SARS-CoV-2 sequences from different geographical regions to those from other viruses, such as SARS and MERS. Although the analyses revealed shared human miRNAs targeting the genome of these viruses, unique miRNAs were observed for SARS-CoV-2. The prediction analysis conducted by Sarma *et al.* (2020), identified 22 potential miRNAs from five genomes of SARS-CoV-2 linked with 12 human miRNAs. Finally, a comparison between the host miRNA binding profiles on 67 different SARS-CoV-2 genomes from 24 different countries revealed miRNAs associated with increased death rates of COVID-19. Recently, Centa *et al.* (2021) reported a significant association in the experimental expression analysis of two miRNAs, miR-26a-5p and miR-29b-3p, with the expression levels of inflammatory markers, such as IL-4, IL-6 and IL-8, in post-mortem lung cells of COVID-19 patients (Centa *et al.*, 2021). These results showed the direct impact of miR deregulation in the endothelial dysfunction and inflammatory response in patients with SARS-CoV-2 infection and acute respiratory injuries.

Among the most common pathways and gene networks affected by the human-virus miRNA interactions are the ones associated with the *ACE2* and *TMPRSS2* genes (Arisan *et al*., 2020; Ghafouri-Fard *et al*., 2020; Hoffmann *et al*., 2020; Lukassen *et al*., 2020; Nersisyan *et al*., 2020; Paniri *et al*., 2021). The miRNAs that regulate the expression of these genes were deregulated in several cardiovascular and pulmonary diseases (Kohlstedt *et al*., 2013; Hu *et al*., 2014; Bao *et al*., 2015; Chen *et al*., 2015), such as the ones developed by many COVID-19 patients. These findings support miRNAs’ role in the development and progression of endothelial and vascular diseases (Ovchinnikova *et al*., 2015; Vegter *et al*., 2017). Taken together, the data presented above show the role of miRNAs in modulating the immune- and other host response-related processes of SARS-CoV-2 infection, suggesting that they can be considered genetic factors for the observed differences in the response of the patients to the infection and in the severity of the disease. As the rich and valuable information obtained through *in silico* analysis becomes increasingly available, additional predictive viral-host miRNAs interactions are expected to be identified, which can lead to the potential identification of miRNAs as therapeutic targets for COVID-19 (Fernández-Hernando and Suárez, 2018; Prestes *et al*., 2020).

In the context of a pandemic, the polymorphisms as well as rare variants that impact disease susceptibility become quantitatively important since millions of people may be infected. Therefore, the knowledge of the genetic variation, at both individual and population levels, may further improve our understanding of the SARS-CoV-2 transmission and pathogenesis, enabling the identification of individuals at high risk of infection and subsequent disease sequelae. More broadly, this may provide valuable information for drug design and vaccine development (Sironi *et al*., 2020).

## Molecular approaches for therapeutic interventions

The use of molecular tools, such as RNA interference (RNAi) is being considered in the search for treatment of COVID-19. The RNAi can directly disrupt the production of viral and/or host proteins involved in SARS-CoV-2 infection, therefore allowing the development of challenging but promising novel therapeutic approaches, which potentially result in specific depletion of key proteins involved in COVID-19 pathogenesis. The RNAi technology itself is simple; it consists of the use of synthetic short interfering RNAs (siRNAs), which can be directly introduced into the cell cytoplasm where they will trigger the degradation of specific mRNA targets. The FDA approval of the first drug based on siRNA (Patisiran), used to treat nerve damage caused by a genetic disease, is encouraging (Uludağ *et al*., 2020). The former studies focused on SARS-CoV-1 infection may guide the work in the current SARS-CoV-2 pandemic. Although RNAi can be directed against any protein, targeting essential viral proteins, such as S, E, M, and N proteins might represent more specific and efficient strategies. In the initial studies applying RNAi against SARS-Co-1, many efforts were performed with the use of siRNAs directed to the S-protein (Qin *et al*., 2004; Zhang *et al*., 2004; Wu *et al*., 2005), the Leader sequence (Li W *et al*., 2005), the non-structural protein 1 (Ni *et al*., 2005), the nucleocapsid N-protein (Zhao *et al*., 2020), the RpRp (He *et al*., 2003; Lu *et al*., 2004) and the E-protein (Meng *et al*., 2006) among others, and obtained considerable success in reducing viral load. Thus, RNAi technology warrants further exploration in order to verify its potential as an alternative strategy for SARS-CoV-2 infection treatment. Recently, several investigators suggested resume efforts focused on this direction (Asha *et al*., 2018; Ghosh *et al*., 2020).

## Pharmacological interventions in cellular and animal models

In order to evaluate potential therapeutic intervention approaches, some strategies focused on ACE2, TMPRSS22, and S protein will be reported. Most of them use inhibitors to reduce the infection rate and the hypertensive and pro-inflammatory effects of Angiotensin II.

### Angiotensin II-converting enzyme (ACE2) receptor inhibitors

ACE2 inhibition has been suggested as a promising approach to attenuate the damage in lung cells caused by SARS-CoV-2 infection (Lopes *et al*., 2020). Captopril, enalapril, losartan and valsartan, which are all ACE2 antagonists, seem to inhibit the receptor and were able to avoid pneumonia caused by SARS-CoV-2 infection (Zhou *et al*., 2020). Further, docking assays and crystallography analysis of virus’ receptor (Benítez-Cardoza and Vique-Sánchez *et al*., 2020; Xia *et al*., 2020) are being explored to support the development of new inhibitory compounds (Tai *et al*., 2020; Yan *et al*., 2020) and small peptides that potentially prevent the interaction between the SARS-CoV-2 S protein and ACE2 (Xiu *et al*., 2020).

### Subunit protein TMPRSS2 Inhibitors

Nafamostat and camostat are serine proteases inhibitors proved to interfere *in vitro* with protein-mediated fusion of SARS-CoV-2 and the host cell (Kang *et al*., 2015; Yamamoto M *et al*., 2020). Camostat can also inhibit TMPRSS2 in the human lung cells infected with SARS-CoV-2 (Hoffmann *et al*., 2020). Clinical trials have been conducted to evaluate the efficacy and safety of camostat mesilate in treating COVID-19. Among those trials is possible to highlight some examples in which the drug was used alone NCT04583592 (CAMELOT, USA); NCT04608266 (CAMOVID, France); NCT04625114 (Belgium); NCT04321096 (Denmark); NCT04470544 (RECOVER, USA) or in association with other drugs NCT04652765 (USA); NCT04750759 (NICCAM; Germany); NCT04355052 (Israel); NCT04662086 (USA); NCT04644705 (Germany) and NCT04518410 (USA). Camostat mesilate are also being studied in several others intervention protocols (NCT04455815, England); NCT04662073 (USA); NCT04530617 (Mexico); NCT04662086 (COPPS study, USA); NCT04374019 (USA) and NCT04518410 (ACTIV-2 study, USA). However, results from all those trials have not been published yet.

VeroE6 cells are a well-known *in vitro* model system that produces high virus titers and displays visual cytopathic effects associated with viral infections. These cells are commonly used in in vitro antiviral assays, including for coronavirus (Matsuyama *et al*., 2010, 2020; Fintelman-Rodrigues *et al*., 2020; Unal *et al*., 2021). Past studies demonstrated that the messenger RNA expression level of TMPRSS2 in VeroE6/TMPRSS2 cells is ~10-fold higher than in normal human lung tissue and other human cell lines. SARS-CoV-2 uses the same receptor, ACE2, as SARS-CoV, and ACE2 expression is very high in VeroE6 cells (Matsuyama *et al*., 2020). In addition, recent studies verified that human Caco-2 colon epithelial cells as well as the lung cell line A549 stably expressing ACE2 and TMPRSS2 (Grobe *et al.*, 2021).

Bromhexine and its metabolite ambroxol are mucolytic drugs that inhibit TMPRSS2, frequently used as a mucolytic agent in respiratory diseases. *In vitro* studies have shown that these drugs hamper the TMPRSS2 effect to activate a zymogen precursor of tissue plasminogen activator and ameliorate the cytokine storm induced by SARS-CoV-2 (Beeh *et al*., 2008; Furgała-Wojas *et al*., 2020). Clinical studies have been carried out using bromhexine (NCT04273763; NCT04355026 and NCT04340349), and preliminary results from NCT04405999 demonstrated that prophylaxis using this drug reduced the rate of symptomatic COVID-19. Aprotinin, enzalutamide, genistein, and estradiol are examples of others TMPRSS2 inhibitors, which were active in vitro using different cell types, however, informations about such effect in vivo are still missing (Royston, 2015; Bestle *et al*., 2020; Wang *et al*., 2020). 

### Furin protease inhibitors

After binding to the ACE2 receptor, the S-protein must be cleaved by the host protease furin for priming the S2 fusion machinery for triggering the fusion of viral and host cell membranes (Bosch *et al*., 2004). Once furin processing is a required step for membrane fusion, furin inhibition could effectively reduce SARS-CoV-2 cell entrance in host cells (Shang *et al*., 2020). Darinaparsin, a currently used anticancer drug, showed a high binding-affinity to furin and could be a hopeful therapy approach for SARS-CoV-2 infection (Chowdhury *et al*., 2020). Estradiol and vitamin D were also able to affect furin’s activity in rat, mouse, and human cells (Glinsky, 2020). The treatment with Vitamin D is still controversial, while some studies have found negative correlation between vitamin D levels and COVID-19 cases (Ilie *et al*., 2020) other hypothesis an alleviation on lung inflammation caused by SARS-CoV-2 because vitamin D seems upregulating ACE2 human receptor and decreasing inflammatory cytokines (Xiao *et al*., 2021).Since, Vitamin D is known to enhance the rate of melanin synthesis; and this may concurrently regulate the expression of furin expression both vitamin D and melanin may have significant impact in management of COVID-19 (Paria *et al*., 2020). Additionally irisin, luteolin, and nafamostat have demonstrated inhibitory activity against furin (Peng *et al*., 2017; de Oliveira *et al*., 2020; Yamamoto M *et al*., 2020). Thus, several known compounds have shown a favorable potential to attack this critical step of SARS-CoV-2 entrance in host cells and reduce infection effectiveness.

### Fusion proteins inhibitors

The development of membrane fusion inhibitors prevents the specific fusion of the viral S2 protein domain, blocking the delivery of viral genetic material into the host cell (Yan *et al*., 2020). The EK1 peptide was able to inhibit SARS-CoV-2 fusion and a novel modified peptide (EK1C4) showed an even higher inhibitory activity against the viral membrane fusion pathway (Xia *et al*., 2020). Lipopeptides (IPB01 and IPB02), designed on the basis of the S-protein S2 fusion domain, demonstrated the ability to inhibit SARS-CoV-2 fusion to host cells (Zhu Y *et al*., 2020). Imatinib might also be involved in the blockage of membrane fusion during coronavirus infection (Sisk *et al*., 2018). 

### Main protease inhibitors

More than four thousand approved commercial drugs were screened *in silico* as potential main protease (Mpro) inhibitors of SARS-CoV-2 infection (Biembengut and de Souza, 2020; Jiménez-Alberto *et al*., 2020). The results evidenced the potential use of several of them in COVID-19 treatment. Drug design recognized the Michael acceptor inhibitor N3 as a potent and irreversible inhibitor of SARS-CoV-1 Mpro (Yang *et al*., 2005). *In vitro* experiments verified that it also inhibited SARS-CoV-2 replication in Vero cells (Jin *et al*., 2020). Furthermore, chemical modifications of Mpro inhibitory groups caused a pronounced lung tropism in mice (Khan S *et al*., 2021, Zhang *et al*., 2021). Peptidomimetic aldehydes also inhibited SARS-CoV-2 replication in Vero E6 cells and showed low toxicity in Sprague-Dawley rats and Beagle dogs (Dai *et al*., 2020). Several natural compounds were also identified as inhibitor candidates of Mpro (Gentile *et al*., 2020; Gurung *et al*., 2020; Khan S *et al*., 2021; Olubiyi *et al*., 2020).

### RNA-dependent RNA polymerase (RpRp) inhibitors

The RDPD can also be a target for pharmacological intervention directed to specifically hinder the function of this enzyme complex (Zhu W *et al*., 2020). A known candidate is favipiravir, which binds to the catalytic domain of RDPR hindering nucleotide inclusion during RNA synthesis (Furuta *et al*., 2017). Some drugs such as ribavirin, remdesivir, sofosbuvir, galidesivir and tenofovir are good candidates as inhibitors of the RNA-polymerase mediated replication (Elfiky, 2020; Soufi and Iravani, 2021). Ribavirin and favipiravir were able to restrain the SARS-CoV-2 RpRp enzymes (Huang *et al*., 2020). Buonaguro *et al*. (2020) described that some commercial drugs with inhibitory activity against the RpRp, including NHC EIDD1931, have suppressed SARS-CoV-2 replication *in vitro* and a preclinical animal model, revealing this pathway as a promising target for therapeutic intervention. 

### Nanotechnology to boost pharmacological therapy

Nanotechnology-based approaches can provide specific drug delivery, enhanced drug bioavailability, low toxicity and improved antiviral activity. Carbon quantum dots inhibited the replication of the human coronavirus (Łoczechin *et al*., 2019). Diphyllin loaded polymeric nanoparticles demonstrated targeted inhibition of the S protein from the feline coronavirus (Hu *et al*., 2017). Glutathione-capped Ag2S nanoclusters also showed antiviral properties by obstructing viral RNA synthesis and budding of porcine epidemic diarrhea virus (PEDV) as a model of coronavirus (Du *et al*., 2018).

## Clinical trials for drug repurposing

Drug repurposing or repositioning is a strategy for identifying new applications for approved or investigational drugs outside the first medical indication (Ashburn and Thor, 2004). Given the high decline rates, high costs, and slow new drug discovery and development’s timeframe, repurposing drugs is frequently becoming an attractive proposition. The rationale is that most of the process includes preclinical tests, safety assessment, and, in some cases, the development of the formulation has already been achieved. Besides, the risk of failure and the timeframe for drug development are almost non-existent (Pushpakom *et al*., 2019).

Until April 2021, more than 5,000 clinical trials were being performed worldwide, evaluating antivirals, corticosteroids, antibiotics, among other drugs against COVID-19 as summarized in Table 1. In the present review, we focus on studies published in journals where publication only occurs after the peer-review process. Here, we emphasize hydroxychloroquine (HCQ), chloroquine, and dexamethasone clinical trials.

Hydroxychloroquine is used to treat malaria, rheumatoid arthritis, and lupus. Some studies point to its antiviral activity against the human immunodeficiency virus (HIV), inhibiting the entry of the virus in host cells and promoting post-translation alteration of newly synthesized proteins via glycosylation inhibition (Rosa and Santos, 2020). Hydroxychloroquine was tested in a retrospective multicenter cohort study of 1438 patients with laboratory confirmation of SARS-CoV-2 infection admitted to 25 hospitals. Four different treatments were evaluated, (1) hydroxychloroquine and azithromycin, (2) hydroxychloroquine, (3) azithromycin, and (4) neither of these drugs. Initially, this study showed that patients who received hydroxychloroquine and azithromycin had a higher incidence of heart failure than the group without treatment. Furthermore, no significant reduction of mortality in the groups of patients receiving any of the treatments compared with the non-treated group (Rosenberg *et al*., 2020).

**Table 1:** Clinical trial for the treatment of COVID-19 with five drugs approved to treat other diseases.

Drug	Participants	Design	Intervention	Conclusion	Reference
Chloroquine	Adult patients who were hospitalized with severe acute respiratory syndrome coronavirus 2 (SARS-CoV-2) infection	Parallel, double-masked, randomized, phase IIb clinical trial	Patients were allocated to receive high-dosage (ie, 600 mg twice daily for 10 days) or low-dosage (ie, 450 mg twice daily on day 1 and once daily for 4 days)	The preliminary outcomes suggest that the higher chloroquine dosage should not be recommended for critically ill patients with COVID-19 because of its potential safety hazards	Borba *et al*., 2020
Hydroxychloroquine	Adults who had household or occupational exposure to someone with confirmed Covid-19	Randomized, double-blind, placebo-controlled trial	Within 4 days after exposure, participants receive either placebo or hydroxychloroquine (800 mg once, followed by 600 mg in 6 to 8 hours, then 600 mg daily for 4 additional days)	Hydroxychloroquine did not prevent illness compatible with Covid-19 or confirmed infection when used as postexposure prophylaxis within 4 days after exposure	Boulware *et al*., 2020
Hydroxychloroquine	Symptomatic, nonhospitalized adults with laboratory-confirmed COVID-19 or probable COVID-19 and high-risk exposure within 4 days of symptom onset.	Randomized, double-blind, placebo-controlled trial	Oral hydroxychloroquine (800 mg once, followed by 600 mg in 6 to 8 hours, then 600 mg daily for 4 more days) or masked placebo.	Hydroxychloroquine did not substantially reduce symptom severity in outpatients with early, mild COVID-19.	Skipper *et al*., 2020
Remdesivir	Adults admitted to hospital with laboratory-confirmed SARS-CoV-2 infection, with an interval from symptom onset to the enrolment of 12 days or less, and radiologically confirmed pneumonia.	Randomised, double-blind, placebo-controlled, multicentre trial	Patients were randomly assigned in a 2:1 ratio to intravenous remdesivir (200 mg on day 1 followed by 100 mg on days 2-10 in single daily infusions) or the same volume of placebo infusions for 10 days. Patients were permitted concomitant use of lopinavir-ritonavir, interferons, and corticosteroids.	Remdesivir was not associated with statistically significant clinical benefits	Wang *et al*., 2020
Remdesivir	Adults who were hospitalized with Covid-19 and had evidence of lower respiratory tract infection	Double-blind, randomized, placebo-controlled trial	Patients were randomly assigned to receive either remdesivir (200 mg loading dose on day 1, followed by 100 mg daily for up to 9 additional days) or placebo for up to 10 days.	Remdesivir was superior to placebo in shortening the time to recovery in adults who were hospitalized with Covid-19 and had evidence of lower respiratory tract infection	Beigel *et al*., 2020
Lopinavir and Ritonavir	Hospitalized adult patients with confirmed SARS-CoV-2 infection	Randomized, controlled, open-label trial	Patients receive either lopinavir-ritonavir (400 mg and 100 mg, respectively) twice a day for 14 days, in addition to standard care, or standard care alone	In hospitalized adult patients with severe Covid-19, no benefit was observed with lopinavir-ritonavir treatment beyond standard care	Cao B *et al*., 2020
Dexamethasone	Hospitalized adult patients with confirmed SARS-CoV-2 infection	Randomized, controlled, open-label trial	Patients receive oral or intravenous dexamethasone (at a dose of 6 mg once daily) for up to 10 days or to receive usual care alone	In patients hospitalized with Covid-19, the use of dexamethasone resulted in lower 28-day mortality among those who were receiving either invasive mechanical ventilation or oxygen alone	RECOVERY Collaborative Group, 2020
Ivermectin	Patients with non-severe COVID-19 and no risk factors for severe disease	Randomized, double-blind, placebo-controlled trial	Patients were randomized 1:1 to receive ivermectin, 400 mcg/kg, single dose (n = 12) or placebo (n = 12).	Among patients receiving a single 400 mcg/kg dose of ivermectin within 72 h of fever or cough onset there was no difference in the proportion of PCR positives.	Chaccour *et al*., 2021
Nitazoxanide	Adult patients presenting up to 3 days after onset of Covid-19 symptoms	Multicenter, randomised, double-blind, placebo-controlled trial	Patients were randomised 1:1 to receive either nitazoxanide (500 mg) or placebo, TID, for 5 days.	Symptom resolution did not differ between nitazoxanide and placebo groups after 5 days of therapy.	Rocco *et al*., 2020

A randomized multicenter study involving 150 patients with moderate-stage COVID-19 in two arms, with or without hydroxychloroquine treatment, found no difference in the evolution of patients who used this drug or not. However, adverse effects related to the use of hydroxychloroquine were reported (Tang W *et al*., 2020). Corroborating this result, Mercuro *et al*. (2020) showed, in a cohort study of 90 patients with COVID-19, that individuals using hydroxychloroquine had an increased risk QT interval prolongation. Also, in a randomized study of patients with severe COVID-19, a high dose of chloroquine alone or with azithromycin/oseltamivir was not recommended due to potential safety hazards related to QT prolongation and increased lethality (Borba *et al*., 2020). A randomized, double-blind, placebo-controlled study tested hydroxychloroquine as post-exposure prophylaxis and concluded that it did not significantly reduce the severity of symptoms in outpatients presenting mild and early COVID-19 (Boulware *et al*., 2020).

The RECOVERY study compared a variety of possible treatments with the usual care in patients hospitalized with COVID-19. The authors examined the daily use of 6 mg of dexamethasone for ten days (2104 patients) versus usual care alone (4321 patients). The preliminary results indicated lower 28-day mortality among patients receiving invasive mechanical ventilation or oxygen alone, but not among those who did not receive respiratory support at randomization (RECOVERY Collaborative Group, 2021).

Ivermectin has been recently proved, in an *in vitro* experiment, to produce reduction in the RNA of SARS CoV-2 at 48 h of its single addition (Caly *et al*., 2020). Among patients with non-severe COVID-19 and no risk factors for severe disease receiving a single 400 mcg/kg dose of ivermectin, Chaccour and colleagues (2021) have found no difference in the proportion of PCR positives. There was however a marked reduction of self-reported anosmia/hyposmia, a reduction of cough and a tendency to lower viral loads and lower IgG titers which warrants assessment in larger trials.

Nitazoxanide, a clinically approved and commercially available antiparasitic drug, has been found to have broad-spectrum antiviral activity, including against coronaviruses, influenza viruses, and hepatitis B and C viruses (Amadi *et al*., 2002). In patients with mild Covid-19, symptom resolution did not differ between nitazoxanide and placebo groups after 5 days of therapy. However, early nitazoxanide therapy was safe and reduced viral load significantly (Rocco *et al*., 2021).

Besides inflammation, COVID-19 patients may present hypercoagulability, characterized by elevation of fibrinogen levels and D-dimers, and may develop disseminated intravascular coagulation (DIC) (Helms *et al*., 2020; Tang *et al*., 2020). Evidence confirms that thrombotic events are associated with higher mortality (Helms *et al*., 2020). Therefore, the Brazilian Society of Thrombosis and Hemostasis (BSTH) and the Thrombosis and Hemostasis Committee of the Brazilian Association of Hematology, Hemotherapy, and Cellular Therapy (ABHH) recommend that all patients hospitalized for suspected or confirmed COVID-19 should receive pharmacologic thromboprophylaxis in the absence of absolute contraindications.

## Immunotherapies: driving the immune response against SARS-CoV2

### Anti-Interleukin 6

Considered one of the most potent cytokines of the inflammatory response, and due to its pleiotropic activity, IL-6 mediates a series of physiological functions, including proliferation, differentiation, activation, and survival of immune response cells (Scheller *et al*., 2011; Tanaka and Kishimoto, 2014; Schaper *et al*., 2015; Murakami *et al*., 2019). Synthesized mainly by lymphocytes, monocytes, and macrophages (Scheller *et al*., 2011; Schaper and Rose-John*,* 2015), as well as stimulated by other cytokines, especially IL-1 and TNF-α (Garbers *et al*., 2012), IL-6 is directly involved in the exacerbation of inflammation (Scheller *et al*., 2011), known as a “hyper-inflammatory state”, which causes intense acute lung injury in severe COVID-19 patients, which can progress to acute respiratory distress syndrome (ARDS) (Swaroopa *et al*., 2016). In an attempt to eliminate SARS-COV-2, this exacerbated and continuous inflammatory reaction, also named “cytokine storm”, essentially has a positive feedback between proinflammatory molecules (mainly IL-6 and TNF-α) and lymphocytes, and also natural killer cells and macrophages (Huang *et al*.,2020; McGonagle *et al*., 2020; Mehta *et al*., 2020; Pedersen and Ho, 2020).

To stop this inflammatory process that is harmful to the patient, some studies (Wu R *et al*., 2020; Xu *et al*., 2020; REMAP-CAP Investigators, 2021) have shown that blocking (tocilizumab or sarilumab) of IL-6 functions promotes a significant clinical improvement and better prognosis for COVID-19 patients with ARDS. Among the main benefits of this treatment, stand out: the reappearance of normal temperature, improvement of oxygenation, reduction of lung injuries, and the return of a healthy percentage of peripheral lymphocytes (Zhang *et al*., 2021). Although basic science suggests rationale for administration of IL-6 receptor antagonists to patients with COVID-19, the clinical evidence regarding the efficacy and safety of tocilizumab remains observational only, according to Cortegiani *et al*. (2021), who investigated 3 indirect pre-clinical and 28 clinical studies. Another difficulty for developing countries is the high cost of this drug.

### Convalescent plasma and neutralizing antibodies-based therapies

Neutralizing antibodies (Nabs) represent an immediate possibility to solve SARS-CoV-2 infection. Therefore, therapy-based studies have also focused on this approach. Nabs target the proteins of the viral surface, impairing its attachment to host cells. Therefore, the ACE2 receptor-binding domain S1 of the SARS-CoV-2 S protein has been pointed out as a major target for Nabs-based strategies by several *in vitro* and *in vivo* models (Duan *et al*., 2020; Wang *et al*., 2020; Wrapp *et al*., 2020; Wu R *et al*., 2020; Zeng *et al*., 2020). 

In this context, convalescent plasma-based therapies are potential strategies to treat SARS-CoV-2 infection, since patients recovered from COVID-19 can present high levels of Nabs (Chen L *et al*., 2020). Historically, passive immunotherapy through the collection and transfusion of convalescent plasma, was first used in the late 19th century (Simon, 2007; Marano *et al*., 2016). During the Spanish flu, the use of these immune derivatives showed effective clinical potential (Bogardus, 1919), reducing the mortality (Luke *et al*., 2006). More recently, convalescent plasma was used during the H1N1 influenza pandemic in 2009 and 2013 during the Ebola outbreak in West Africa. However, the antibody levels in COVID-19 convalescent plasma are highly variable, and assays to determine the effective antibody titers remain limited (Brown and McCullough, 2020).

Some studies have demonstrated a reduction in viral load in COVID-19 patients treated with convalescent plasma (Ahn *et al*., 2020; Duan *et al*., 2020; Shen C *et al*., 2020; Ye *et al*., 2020; Zhang *et al*., 2021). Almost all patients showed improvement in the clinical, laboratory and imaging parameters. However, it was not possible to attribute the favorable clinical response to convalescent plasma, as the multiplicity of drugs used and the lack of controls prevented this conclusion (Ye *et al*., 2020).

### Anti-complement approaches

The inhibition of critical inflammatory components of the complement cascade seems to be very useful because, at the same time that it blocks the adaptive immune response, it can control the tissue damage associated with the cytokine storm in severe cases of COVID-19 (Chauhan *et al*., 2020). This strategy was recently tested during three weeks in ten patients treated with a combination of ruxolitinib, a JAK1/2 inhibitor, and eculizumab, an anti-C5a complement monoclonal antibody. The results showed improved lung function and decreased circulating D-dimer levels (Giudice *et al*., 2020). Interestingly, some studies have proposed that complement blockade might be of benefit in severe COVID-19; however, several risk factors for such infections were related following eculizumab administration (Diurno *et al*.,2020; Laurence *et al*., 2020). This medicine is still being investigated in clinical trials (NCT number: 04288713 and NCT number: 04346797) for the treatment of moderate to severe pneumonia related to COVID-19.

### Main vaccines against Sars-CoV-2 available

CoronaVac is produced by the Chinese company Sinovac Biotech. The vaccine uses the inactivated Sars-CoV-2 virus in its formulation as well as other vaccines that are under development, such as BBIBP-CorV and BBV152 (Zang *et al.*, 2021). The vaccine passed Phase III clinical trials in Brazil, Chile, Indonesia, the Philippines, and Turkey. CoronaVac does not need to be frozen, and both the vaccine and raw material for formulating the new doses could be transported and refrigerated at 2-8 ° C, temperatures at which flu vaccines are kept (Sinovac Biotech). 

Several results from CoronaVac’s Phase III demonstrate positive results regarding its effectiveness. A study in Chile found it 67% effective against symptoms, reduced hospitalizations by 85%, intensive care visits by 89%, and deaths by 80%. In Brazil, it showed 50.7% effectiveness at preventing symptomatic infections and 83.7% effective in preventing mild cases needing treatment. Effectiveness against symptomatic infections increased to 62.3% with an interval of 21 days or more between the doses (Mallapaty, 2021). Final Phase III results from Turkey announced on 3 March 2021 showed an effectiveness of 83.5% (Riad *et al*., 2021).

On January 22, 2021 the Brazil’s health regulatory agency (Anvisa) granted the first CoronaVac vaccine registration against COVID-19, for emergencial use in Brazil. The immunizer from the Sinovac/Butantan Laboratory had its safety, quality and effectiveness checked and attested by Anvisa’s technical team (https://vacinacovid.butantan.gov.br/). 

The vaccine produced by the pharmaceutical company AstraZeneca in conjunction with the University of Oxford has become a wide option in the fight against SARS-CoV-2. It uses a chimpanzee common cold viral vector known as ChAdOx1, which expresses the gene that allows human cells to produce the SARS-CoV-2 spike protein (AstraZeneca). Between April 23 and Nov 4, 2020, 11 636 participants from UK and Brazil were included in the interim primary effectiveness analysis. In participants who received two standard doses, vaccine effectiveness was 62.1% and in participants who received a low dose followed by a standard dose, effectiveness was 90,0%. Overall vaccine effectiveness across both groups was 70,4% (Voysey *et al*., 2021). 

On March 12, 2021 the Anvisa authorized the distribution of the AstraZeneca / Oxford vaccine in Brazil. The immunizer produced in Brazil within Fiocruz Institute had its safety, quality and effectiveness checked and attested by Anvisa’s technical team (Ministério da Saúde, 2021c).

Another vaccine against COVID-19 similar to AstraZeneca’s is produced by the pharmaceutical company Janssen. It is known as JNJ-78436735 or Ad26.COV2.S. The viral agent used as a vector is adenovirus 26. Initially, the Janssen vaccine was shown to induce antibodies against SARS-CoV-2 in 90% of people after the first dose. Just one dose of vaccine was 66% effective in preventing moderate to severe COVID-19 and 100% effective in preventing COVID-19-related hospitalization and death (Livingston *et al*., 2021).

The Pfizer/BioNTech Vaccine is a lipid nanoparticle-formulated, nucleoside-modified mRNA encoding the prefusion spike glycoprotein of SARS-CoV-2. This vaccine has been recommended to people 16 years of age and older, with a dose of 30 μg (0.3 mL) IM. The vaccination requires two shots given 21 or more days apart. Anti-SARS-CoV-2 antibodies persist for at least 119 days after the first vaccination and prevention of the SARS-COV-2 infection is 95% effective (Oliver *et al*., 2020; Meo *et al*., 2021). On December 11, 2020, the US Food and Drug Administration (FDA) authorized the emergency use of the Pfizer-BioNTech COVID-19 vaccine (FDA, 2020).

On February 23, 2021 the Anvisa granted the first registration of the Pfizer/BioNtech vaccine for widespread use in the Americas. The vaccine had its safety, quality and effectiveness checked and attested by Anvisa’s technical team of servers (Ministério da Saúde, 2021a).

The Russian Institute Gamaleya developed Sputnik V (Gam-COVID-Vac), an adenovirus-based candidate vaccine against COVID-19e The Sputnik V vaccine consists of two replication-defective recombinant adenoviruses: type 26 (rAd26-S) and type 5 (rAd5-S), both carrying the gene for the SARS-CoV-2) spike glycoprotein (Logunov *et al*., 2020). The results of phase I-II studies indicated good immunogenicity and safety, however, only 38 volunteers were enrolled for each of the two formulations (frozen and lyophilized) (Logunov *et al.*, 2020). Recent interim results of a Sputnik V phase 3 trial in a large cohort indicated 91.6% effectiveness against COVID-19 and lack of adverse vaccination-related adverse effects (Logunov *et al.*, 2021).

However, the development of the Sputnik V vaccine has been criticized for unseemly haste, corner cutting, and an absence of transparency (Balakrishnan, 2020; Cohen, 2020; Bucci *et al*., 2021). Serious concerns regarding interim results from the phase III trial were also raised (Bucci *et al.*, 2021). Data sharing is one of the cornerstones of research integrity, yet Logunov *et al.* (2021) stated that raw data will not be shared before the trial is completed. Among the concerns raised are: the full study protocol has not been made publicly available; the clinical and laboratory criteria used to determine suspected COVID-19 were not informed; the data, numerical, and statistical significance results reported showed major inconsistencies (Bucci *et al.*, 2021).

On April 27, 2021 the Anvisa announced that the import of the Sputnik V vaccine was not approved for use in Brazil. According to the agency, after evaluation, flaws in the development and production of the immunizing agent would have been found (Ministério da Saúde, 2021b). The concerns are similar to those now reported in May 2021 by Bucci *et al*. (2021).

## Final considerations

Twenty months after the first Covid-19 notifications, more than 170 million individuals were infected worldwide with SARS-CoV-2, and around 3.5 million deaths occurred. Unlike the period of the last great pandemic that occurred at the beginning of the past century, the COVID-19 pandemic occurs at a time of significant scientific and technological advances in biomedical sciences, which, in theory, could be applied immediately in the control and treatment of patients. However, no drug or vaccine has yet been specifically approved for COVID-19. Therapeutic intervention approaches used successfully in other infectious agents need an in-deep investigation directed to the specific infection mechanism of the SARS-CoV-2 and the unique COVID-19 physiopathology. Among the available therapeutic approaches, such as vaccines, target inhibitors, and new drugs, the drug repurposing already approved by the FDA has been shown to be an efficient short-term alternative, mainly due to its low cost and prompt application to patients. This strategy considers the knowledge of the molecular basis of the disease. As a result of the global task force to control the COVID-19 pandemic, a new intervention was introduced by Garvin *et al*. (2020), who blamed the bradykinin storm for the most severe symptoms of COVID-19. The authors point out that many of the symptoms manifested by patients with COVID-19 are similar to other clinical conditions caused by the increase in bradykinin. The strategy would be pharmacologic intervention targeting the renin-angiotensin system to reduce bradykinin levels. In this sense, there exist at least ten approved drugs that might be used to control the severe symptoms of COVID-19.

The genetic variability of molecules that participate in the entry of SARS-CoV-2 into the host cells and, especially, of the numerous molecules involved in the immune responses should be considered for the development of effective therapeutic interventions. Because the frequencies of genetic variants influencing the response to drugs, as well as COVID-19 susceptibility and severity may differ widely among world populations, knowing their distribution is a critical element in seeking strategies to respond to the pandemic. Moreover, understanding the repertoire of viral epitopes that specific HLA allotypes can bind is of great importance for the development of vaccines that can provide protection for most individuals.

Computational modeling and simulations with toxicity analysis scenarios are needed to boost pharmacological interventions and drug repurposing, aiming at potential drugs to reduce viral load, viral clearance, and morbidity and mortality in clinical outcomes (Al-Kofahi *et al*., 2020). New therapeutic agents can be developed by analyzing theoretical structure-activity data in a three-dimensional approach, obtained by recent molecular modeling techniques. Choosing the right dose for a clinical trial requires considering the risk of toxicity and ensuring the best chance of successfully reaching therapeutic targets (Al-Kofahi *et al*., 2020; Dong *et al*., 2020). It is noteworthy that *in vitro* to *in vivo* extrapolations can underestimate or overestimate the real needs of medicines, but it is considered an initial advance.
